# Post-pulmonary tuberculosis lung function: a systematic review and meta-analysis

**DOI:** 10.1016/S2214-109X(25)00105-6

**Published:** 2025-05-21

**Authors:** Sharenja Ratnakumar, Sally E Hayward, Emma K Denneny, Lucy P Goldsmith, Rebecca Evans, William Checkley, Delia Goletti, Catherine W M Ong, Mateusz Gotowiec, Junkai Zhu, Jon S Friedland, Joanna C Porter

**Affiliations:** a UCL Respiratory, University College London, London, UK; b Department of Methodology, Medical University of Warsaw Institute for Infection and Immunity, Warsaw, Poland; c St George's School of Health and Medical Sciences, City St George's University of London, London, UK; d Public Health Research Institute, St George's School of Health and Medical Sciences, City St George's University of London, London, UK; e Division of Pulmonary and Critical Care, Johns Hopkins University, Baltimore, MD, USA; f Center for Global Non-Communicable Disease Research and Training, Johns Hopkins University, Baltimore, MD, USA; g Department of Epidemiology and Preclinical Research, National Institute for Infectious Diseases L Spallanzani-IRCCS, Rome, Italy; h Infectious Disease Translational Research Programme, National University of Singapore, Singapore; i Division of Infectious Diseases, National University Hospital, Singapore; j National University of Singapore Institute for Health Innovation & Technology (iHealthtech), Singapore

## Abstract

**Background:**

Although post-tuberculosis lung disease poses a substantial threat to individuals who have recovered from pulmonary tuberculosis, data showing objective functional impairment in such people are scarce. We did a systematic review and meta-analysis to estimate respiratory impairment after pulmonary tuberculosis disease and examine differences in ventilatory defects.

**Methods:**

We systematically searched Embase, MEDLINE, and CINAHL from Jan 1, 2000, to Dec 13, 2024. We included any study design with data on lung function tests in individuals with a previous diagnosis of pulmonary tuberculosis versus healthy controls. Outcomes extracted from eligible studies included forced expiratory volume in 1 s (FEV_1_), forced vital capacity (FVC), FEV_1_ as a percentage of the predicted value (FEV_1_%), FVC as a percentage of the predicted value (FVC%), and FEV_1_/FVC ratio. Pre-bronchodilator values were preferentially selected. Random effects mean difference models were used when possible and standardised mean difference where it was necessary to standardise to a single scale (ie, FEV_1_%, FVC%, and FEV_1_/FVC ratio). Between-study heterogeneity was estimated with *I*^2^. This study was prospectively registered with PROSPERO (CRD42021248838).

**Findings:**

Of the 5594 publications found, data from 19 studies were included for meta-analyses, reporting on 75 960 individuals of whom 7447 had past pulmonary tuberculosis. All studies reporting absolute values, using various levels of adjustment or standardisation, showed that previous pulmonary tuberculosis had a negative effect across all spirometric values: FEV_1_ –0·41 L (95% CI –0·51 to –0·32, *I*^2^=90·4%), FVC –0·25 L (–0·33 to –0·17, *I*^2^=80·6%), and FEV_1_/FVC ratio –0·37 (–0·54 to –0·19, *I*^2^=92·0%). In those studies, using reference values to derive FEV_1_% and FVC %, prior pulmonary tuberculosis had a pooled standardised mean difference of –0·44 (–0·60 to –0·28, *I*^2^=95·6%) and –0·33 (–0·54 to –0·13, *I*^2^=91·3%), respectively, compared with controls.

**Interpretation:**

People who recover from pulmonary tuberculosis have significantly decreased lung function compared with controls, with FEV_1_ more affected than FVC, giving a mixed obstructive and restrictive picture with predominantly airflow obstruction.

**Funding:**

None.

## Introduction

In 2023 an estimated 10·8 million people were affected by tuberculosis disease, resulting in 1·25 million deaths.^1^ Although treatment of drug-susceptible pulmonary tuberculosis is highly effective at 88%,^1^ microbiological cure is unlikely to represent the end of the illness.^2^ People who recover can be affected by residual pulmonary fibrosis, cavitation, and structural distortion ultimately leading to pulmonary remodelling, which affects respiratory capacity and function.^3^ This situation is further complicated by the rising burden of multidrug-resistant and extensively drug-resistant tuberculosis, resulting in more destructive lung disease which exacerbates complications.^4^ Consequently, respiratory diseases remain the leading cause of excess deaths in people who recover from tuberculosis, among whom mortality is three to six times greater than among their peers in the same location.^5^, ^6^ With an estimated 79 million lives saved through successful tuberculosis treatment since 2000^1^ and a rising life expectancy, this tuberculosis-related lung condition poses a major threat to public health.

Post-tuberculosis sequalae with residual lung damage occurs in about 18–80% of patients^3^ and pulmonary dysfunction increases risk of death from respiratory causes.^6^ A distinct feature of pulmonary tuberculosis is its striking heterogeneity in severity and clinical outcomes between patients. This heterogeneity remains largely unexplained and contributes to the difficulties in accurate estimation of disease burden. The term post-tuberculosis lung disease is increasingly recognised within the tuberculosis research community, highlighting the need for more attention and interventions targeting these long-term sequelae.^7^

Post-tuberculosis lung disease can cause airflow obstruction, restrictive ventilatory defects, and impairment in gas exchange. Recent data, including large population-based studies such as PREPOCOL (Columbia),^8^ PLATINO (South America),^9^ and BOLD (19 global sites),^10^ indicate that tuberculosis contributes substantially to the growing burden of chronic obstructive pulmonary disease. In some low-income and lower-middle-income countries, tuberculosis-associated obstructive pulmonary disease has emerged as a distinct clinical entity, disproportionately affecting younger populations, unlike smoking-associated chronic obstructive pulmonary disease (COPD), which develops later in life.^11^, ^12^

**Research in context**

**Evidence before this study**

An estimated 79 million people have recovered from tuberculosis since 2000, and a growing number of cross-sectional and longitudinal studies have described chronic lung disease and increased mortality among these individuals. However, as data on continuing care beyond tuberculosis treatment are incomplete, the extent of the respiratory impairment among individuals who have recovered from pulmonary tuberculosis is poorly characterised. Before undertaking this systematic review and meta-analysis, we searched MEDLINE, Embase, Scopus, and the Cochrane Library from database inception to March 1, 2024, with the use of search terms including “tuberculosis”, “pulmonary function”, “lung impairment”, “chronic lung disease”, “airflow obstruction”, and “post-tuberculosis sequelae”. We screened for systematic reviews, meta-analyses, and primary studies describing long-term pulmonary outcomes after tuberculosis treatment. Although several primary studies reported post-tuberculosis lung function, we identified no comprehensive systematic reviews or meta-analyses that pooled lung function measures across diverse populations, stratified by tuberculosis status and adjusted for confounders. To our knowledge, this study is the first to provide a global synthesis of spirometric outcomes in individuals who have recovered from pulmonary tuberculosis.

**Added value of this study**

This is a comprehensive systematic review and meta-analysis exploring the extent of lung function impairment in individuals who recovered from pulmonary tuberculosis against healthy populations. A total of 75 960 participants, across five WHO regions, were included for quantitative analysis, of which 7447 had a history of pulmonary tuberculosis disease. We found that, overall, previous pulmonary tuberculosis had a negative pooled effect on all measured spirometric parameters. The condition affects forced expiratory volume in 1 s more substantially than forced vital capacity, in both absolute and percentage of predicted values, thus showing a mixed obstructive and restrictive picture, with a predominant airflow obstruction deficit in people who recovered from pulmonary tuberculosis. Our research provides a new insight into documented respiratory impairment in pulmonary tuberculosis survivors that has been overlooked in previous population studies, which have mainly focused on chronic obstructive pulmonary disease.

**Implications of all the available evidence**

Our study has substantial implications for clinical practice and policy. Post-tuberculosis lung disease is an under-recognised global challenge, with no evidence-based recommendations for investigation and management available. However, this systematic review provides compelling evidence that post-tuberculosis lung disease requires long-term respiratory care, which should be an explicit component of the WHO End Tuberculosis strategy.

However, due to the insufficient number of high-quality controlled trials in this population, no evidence-based recommendations for the investigation and management of post-tuberculosis lung disease are currently available. The WHO End Tuberculosis strategy still focuses on reducing tuberculosis incidence and mortality and makes no mention of post-tuberculosis lung disease.^13^ Furthermore, WHO-recommended tuberculosis registries do not capture data beyond cure.^14^ Only over the last 4 years, a few consensus-based sets of clinical standards for post-tuberculosis lung disease have been published, which are mainly directed towards pulmonary rehabilitation strategies.^15^, ^16^

There has been little comprehensive examination of the evidence base on changes in lung function tests after tuberculosis disease. We conducted a systematic review and meta-analysis to estimate respiratory impairment after pulmonary tuberculosis disease and examine differences in ventilatory defects.

## Methods

### Search strategy and selection criteria

We did a systematic review and meta-analysis, registered with PROSPERO (CRD42021248838), following the University of York Centre for Reviews and Dissemination guidelines^17^ and PRISMA reporting standards.^18^ We searched the medical databases MEDLINE, Embase, and CINAHL from Jan 1, 2000, to Dec 13, 2024, using a Boolean search strategy to combine keywords and subject headings for tuberculosis with those for lung function tests (appendix p 1). Our search strategy started from the year 2000, to coincide with the active implementation of the Global Initiative for Chronic Obstructive Lung Disease, and therefore better capture ventilatory deficits across the studies.^19^ No language restrictions were imposed as we did not want to introduce bias by excluding non-English publications. We also completed bibliographic screening and citation searching (with the use of the Web of Science citation search tool) of the included papers, did bibliographic screening of any existing reviews identified as being of relevance, and consulted independent experts in the field (DG, CWMO, and WC).

All records retrieved during the searches were imported into EndNote and duplicates were deleted. Title and abstract as well as full-text screening was done by pairs of independent reviewers (SR, RE, EKD, MG, JZ, and JCP) with the use of Rayyan (v1.5.3)^20^ and disagreements were resolved through discussion and input of a third reviewer when necessary. Google Translate was used for screening of non-English titles and abstracts, with the plan for professional translation services for full texts when required. Reason for exclusion was noted at the full-text screening stage.

Studies were included if they met all the following criteria: original research papers; included clinical, microbiological, self-reported, or treatment history of pulmonary tuberculosis; had a healthy control population; reported lung function tests as an outcome for participants; and reported or provided enough data to separate outcomes between individuals with previous pulmonary tuberculosis and controls. Studies were excluded if the control had a substantial selection bias, specifically to groups with respiratory disease, or if studies only contained data on secondary outcomes or reported duplicate data. When a population had been reported in several publications, we extracted results from the study with the most extensive exclusion criteria for the healthy control group, particularly if these factors might influence lung function outcomes (eg, acute illness and chronic respiratory conditions). Full selection criteria are outlined in the appendix (p 2).

The primary outcomes extracted were measurable effects on lung function, as identified through at least one lung function test but not limited to: forced vital capacity (FVC), forced expiratory volume in 1 s (FEV_1_), FEV_1_ as a percentage of that predicted (FEV_1_%), FVC as a percentage of that predicted (FVC%), FEV_1_/FVC ratio, flow volume loops, peak expiratory flow, transfer and diffusion factors, and residual volume. Effect measures for the relationship between pulmonary tuberculosis and lung function were extracted when reported in the included studies (eg, relative risk and odds ratios). Secondary outcomes extracted from included studies were any significant radiological findings or data on markers of inflammation.

### Data analysis

Quality appraisal of all included studies was done with the use of the appropriate Joanna Briggs critical appraisal tool for cohort, case-control, or cross-sectional studies.^21^ All papers were given a quality score, with a score of 75% or more representing high quality, more than 50% and less than 75% representing medium quality, and 50% or less low quality. Data extraction and appraisal was carried out in duplicate by two independent reviewers, with disagreements resolved through discussion and input of a third reviewer where necessary. Studies were not excluded based on quality, but information on quality was considered in the synthesis.

We used STATA (version 18)^22^ to calculate effect estimates and associated 95% confidence intervals (CIs). For the outcomes FEV_1_ and FVC, random-effects models were used to calculate mean difference. Random effects standardised mean difference (SMD) models were used for the outcomes FEV_1_%, FVC%, and FEV_1_/FVC ratio. A combined natural log odds ratio was obtained by back transformation. When there were several results reported for a single outcome of interest, we extracted results based on the following preferences: pre-bronchodilator values, data after one episode of treated pulmonary tuberculosis rather than multiple episodes, estimates adjusted for the greatest number of potentially confounding baseline covariates, and data for the longest follow-up period.

Further regression and sensitivity analyses were done in STATA with the use of the metan command.^22^ To assess the robustness of our findings, we repeated all analyses with the use of fixed-effects models and carried out leave-one-out analysis and subgroup analyses according to study characteristics chosen post hoc. Statistical heterogeneity was assessed with the use of the *I*^2^ statistic and p values reported. An *I*^2^ value of 50–75% suggested moderate heterogeneity and 75% or more suggested high heterogeneity.^23^ We assessed publication bias by visual examination of funnel plots and with the use of Egger tests where the analysis included at least the minimum number of studies needed to distinguish chance from real asymmetry.^22^, ^24^

### Role of the funding source

There was no funding source for this study.

## Results

**Figure 1:**
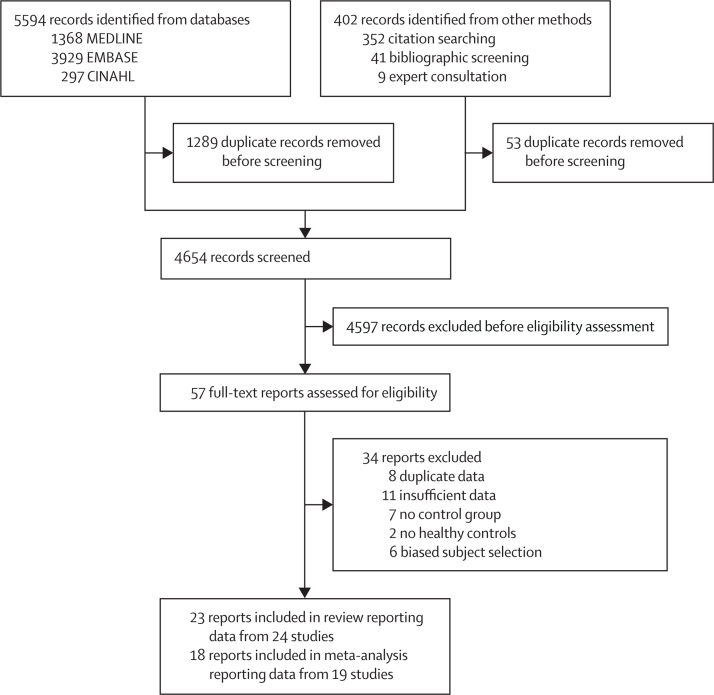
Study selection

Database searches yielded 5594 publications, with 4305 publications carried forward for title and abstract screening after removal of duplicates (figure 1). A further 349 records were identified through bibliography screening and recommendations from experts. The full texts of 57 publications were screened, of which 34 records were excluded. Altogether, 23 publications (reporting data from 24 studies) met the inclusion criteria, with 19 studies included for quantitative synthesis (figure 1). No translations were required. Studies excluded consisted of eight due to duplicates, 11 with insufficient data, seven without control groups, two with no healthy controls, and six with biased selection.

A total of 75 960 participants were included for quantitative analysis, of whom 7447 had a history of pulmonary tuberculosis. The characteristics of these studies are presented in the table. The publications included in the analyses comprised 12 cross-sectional,^9^, ^26^, ^27^, ^28^, ^32^, ^35^, ^36^, ^38^, ^39^, ^40^, ^41^, ^45^ three cohort,^25^, ^47^, ^48^ and four case–controls studies^30^, ^32^, ^42^, ^47^ containing data from 2000 to 2024. Studies were conducted in five WHO regions (appendix pp 4–7), across 22 countries: South Africa (four studies),^25^, ^32^, ^46^, ^47^ Benin (one study),^35^ Sudan (one study),^33^ Malawi (two studies),^27^, ^46^ Uganda (one study),^40^ India (three studies),^31^, ^40^, ^43^ South Korea (two studies),^28^, ^38^ China (two studies),^26^, ^44^ Tibet (one study),^45^ Indonesia (one study),^30^ Peru (two studies),^34^, ^40^ Brazil (one study),^9^ Uruguay (two studies),^9^, ^40^ Mexico (one study),^9^ Chile (two studies),^9^, ^40^ Venezuela (one study),^9^ The Gambia (one study),^41^ Bangladesh (one study),^40^ and Congo (one study),^40^ Argentina (two studies),^29^, ^40^ Egypt (one study),^37^ and Nigeria (one study;^42^ table).

**Table:** Summary of included study characteristics

	**Location**	**Study type**	**Past PTB n (men/women)**	**Control n (men/women)**	**Outcome measure**	**Levels of adjustment or standardisation**	**Study quality**
Hnizdo et al (2020)^25^	South Africa	Cohort	2137 (2137/0)	23 712 (23 712/0)	FEV_1_, FVC, and FEV_1_%	Population of men only, all adjusted for age and height. Predicted values from minors without PTB or pneumoconiosis	73%
Menezes et al (2007)^9^	Brazil, Uruguay, Mexico, Chile, and Venezuela	Cross-sectional	132 (44/88)	5439 (2148/3291)	FEV_1_, FVC, FEV_1_/FVC; FEV_1_%, FVC%; and OR of AFO*	Predicted values from PLATINO reference curves. OR adjusted for sociodemographic, smoking, indoor and occupational pollution, and history of hospitalisation	88%
Lam et al (2010)^26^	China	Cross-sectional	1954 (722/1232)	6112 (1411/4701)	FEV_1_% and OR for AFO†	Predicted values from Chinese reference population. OR adjusted for age, sex education, smoking, biomass, and dust exposure	100%
Fullerton et al (2011)^27^	Malawi	Cross-sectional	19	278	FEV_1_	Adjusted for age, sex, height, cooking material, household location, sleeping location, and economic status	50%
Lee et al (2011)^28^	South Korea	Cross-sectional	294 (184/110)	3393 (1510/1883)	FEV_1_, FVC, FEV_1_/FVC; FEV_1_%, FVC%; and OR for AFO*	Predicted values from KNHANES survey population; OR adjusted for CXR lesion, smoking, sex, and age	75%
Gomez (2012)^29^‡	Argentina	Case–control	25 (12/13)	27 (9/18)	FEV_1_%	Not adjusted	40%
Ralph et al (2013)^30^‡	Indonesia	Prospective and case-control	200 (131/69)	40 (31/9)	FEV_1_	No difference in sex, age, ethnicity, and height between groups. Controls age, sex, height, and ethnicity matched	60%
Dhooria et al (2014)^31^	India	Prospective and case–control	50 (33/17)	50 (31/19)	FEV_1_ and FVC	Controls age and sex matched	90%
Cole et al (2016)^32^	South Africa	Cross-sectional	15	12	FEV_1_/FVC and FEV_1_%	All adjusted for age, sex, current smoking, body mass index, and HIV status	63%
Osman et al (2016)^33^	Sudan	Case–control	136 (99/37)	136 (99/37)	FEV_1_, FVC, and FEV_1_/FVC; FEV_1_% and FVC%; and OR for AFO*	Controls age and sex matched. Predicted values from European Respiratory Society 93′ or adjusted for age and treatment delay	80%
Byrne et al (2017)^34^	Peru	Cohort—two groups (drug-sensitive and MDR)	Drug susceptible 144 (83/61); MDR 33 (19/14)	161 (49/112)	FEV_1_ and FVC; and OR for AFO*	All values adjusted for: age, sex, height, smoking, environmental exposure to tobacco smoke, indoor air pollution, occupational dust, born outside Lima, and presence of atopy	64%
Fiogbe et al (2019)^35^	Benin	Cross-sectional	189 (128/61)	70 (48/22)	FEV_1_ and FVC	Adjusted for age, sex and tobacco and biomass exposure. Controls matched by sex, age, and size	100%
Nightingale et al (2019)^36^	Malawi	Cross sectional	47	1434	FEV_1_, FVC, and FEV_1_/FVC	Adjusted for age, sex, weight, and height	88%
El Sayed El Shourbagy et al (2019)^37^*‡	Egypt	Case–control	20 (10/10)	20 (10/10)	FEV_1_, FVC; FEV_1_%; and FEV_1_/FVC	Controls were age and sex matched	70%
Kim et al (2019)^38^	South Korea	Cross-sectional	1482 (847/635)	14 034 (6013/8021)	FEV_1_, FVC, and FEV_1_/FVC; and FEV_1_% and FVC%	Not adjusted. Predicted values from KNHANES survey population^39^	100%
Kamenar et al (2022)^40^	Argentina, Uruguay, Bangladesh, Uganda, Peru, Congo, India, and Chile	Cross-sectional	332	12 064	FEV_1_ and FVC; and FEV_1_/FVC	All adjusted for sex, smoking, age, education, and biomass fuel use	100%
Nkereuwem et al (2022)^41^	The Gambia	Cross-sectional	52	89	FEV_1_, FVC, and FEV_1_/FVC	Data were standardised using the Global Lung Initiative 2012 African American reference ranges. The African American reference ranges have been validated among African children	88%
Fink et al (2022)^42^‡	Nigeria	Cohort	49	147	FEV_1_, FVC, and FEV_1_/FVC	Values used not adjusted	75%
Shanmugasundaram et al (2022)^43^‡	India	Case–control	10 (5/5)	10 (5/5)	FEV_1_%, FVC%; FEV_1_/FVC; and impulse oscillometry parameters	No difference in age, height, or sex between groups. Predicted values based on reference values from Global Lung Initiative South-East Asian population	70%
Shui et al (2023)^44^	China	Case–control	51 (18/33)	51 (24/27)	FEV_1_%, FVC%, FEV_1_/FVC; and transfer factor, lung volumes, and cardiopulmonary exercise test parameters	BMI and gender-matched to non-smoking healthy controls. Percentage of the predicted values based on patient gender, age, weight, and height	50%
Xing et al (2023)^45^	Tibet and Xiangjiang Uygur autonomous region	Cross-sectional	610 (328/282)	8070 (3945/4125)	FEV_1_, FEV_1_%, FVC, FVC%, and FEV_1_/FVC; small airway dysfunction assessment; and OR for AFO*	Predicted values based on the reference values from the lung function equations for a northeast Asian population. Adjusted OR: adjustments for age, sex, region, education plus history of asthma, exposure to household air pollution and occupation, and smoking status	75%
Martinez et al (2023)^46^‡	South Africa	Prospective cohort	96	972	Tidal breathing measures, respiratory impedance, and lung clearance index	Adjusted infant length, sex, maternal HIV status, maternal smoking during pregnancy, age at visit, and previous lung function measurements	91%
van der Zalm et al (2024)^47^	South Africa	Prospective cohort	50 (19/31)	50 (28/22)	FEV_1_ and FVC; FEV_1_/FVC; and transfer factor and lung volumes	Adjusted for age, sex, height, and ethnicity (other)^48^ with the use of Global Lung Initiative reference	90%

AFO=airflow obstruction. CXR=chest x-ray. FEV_1_=forced expiratory volume in 1 s. FVC=forced vital capacity. FEV_1_%=FEV_1_ as a percentage of the predicted value. FVC%=FVC as a percentage of the predicted value. MDR=multidrug resistant. OR=odds ratio. PTB=pulmonary tuberculosis.

* Defined by FEV_1_/FVC<0·7.

† Defined by FEV_1_/FVC<lower limit of normal.

‡ Not included in meta-analysis.

The average age range of individuals who had recovered from pulmonary tuberculosis was 5–62 years, with many studies skewed towards a younger population (<50 years). One study included children younger than 12 years (appendix pp 4–7).^41^ Altogether 47% of participants were women and many were from upper-middle-income to low-income countries (appendix pp 4–7). Several studies defined a previous diagnosis of pulmonary tuberculosis with confirmed medical histories,^9^, ^25^, ^29^, ^30^, ^31^, ^32^, ^33^, ^35^, ^37^, ^47^, ^48^, ^49^ whereas the remaining studies used either self-reporting^27^, ^36^, ^38^, ^40^ or radiology (appendix pp 4–7).^26^, ^28^, ^38^, ^45^

Three studies used post-bronchodilator values,^33^, ^39^, ^43^ whereas one^31^ did not specify whether pre-bronchodilator or post-bronchodilator values were used, but all were included in analyses. One study^34^ presented two populations with previous pulmonary tuberculosis—drug-sensitive and multidrug-resistant disease. One study included a single participant who was HIV-positive in the healthy control group.^47^ Given the negligible effect on the overall control group, the study was retained in the meta-analysis. Most studies used some level of adjustment or standardisation to process absolute values and reference populations to derive percentage of predicted values (table). The quality of the included studies ranged from fair to high, with three studies noted as low quality (table, appendix pp 8–9).^27^, ^29^, ^44^ Publications were not excluded based on study quality.

All studies consistently observed a negative effect of previous pulmonary tuberculosis on lung function compared with healthy controls across all spirometry measures. Two studies reported on a reduction of transfer factor, lung volumes, and exercise capacity in participants with previous tuberculosis.^44^, ^47^ Xing and colleagues^45^ specifically showed small airways dysfunction was associated with previous pulmonary tuberculosis. Four studies excluded from meta-analysis showed significantly lower FEV_1_^37^, ^43^ and FEV_1_%^29^, ^37^ in individuals with previous pulmonary tuberculosis versus controls. Two studies^29^, ^37^ reported no difference in bronchial responsivity, measured by methacholine challenge testing between groups, although increased bronchial responsivity was found in patients with active tuberculosis.^37^

**Figure 2:**
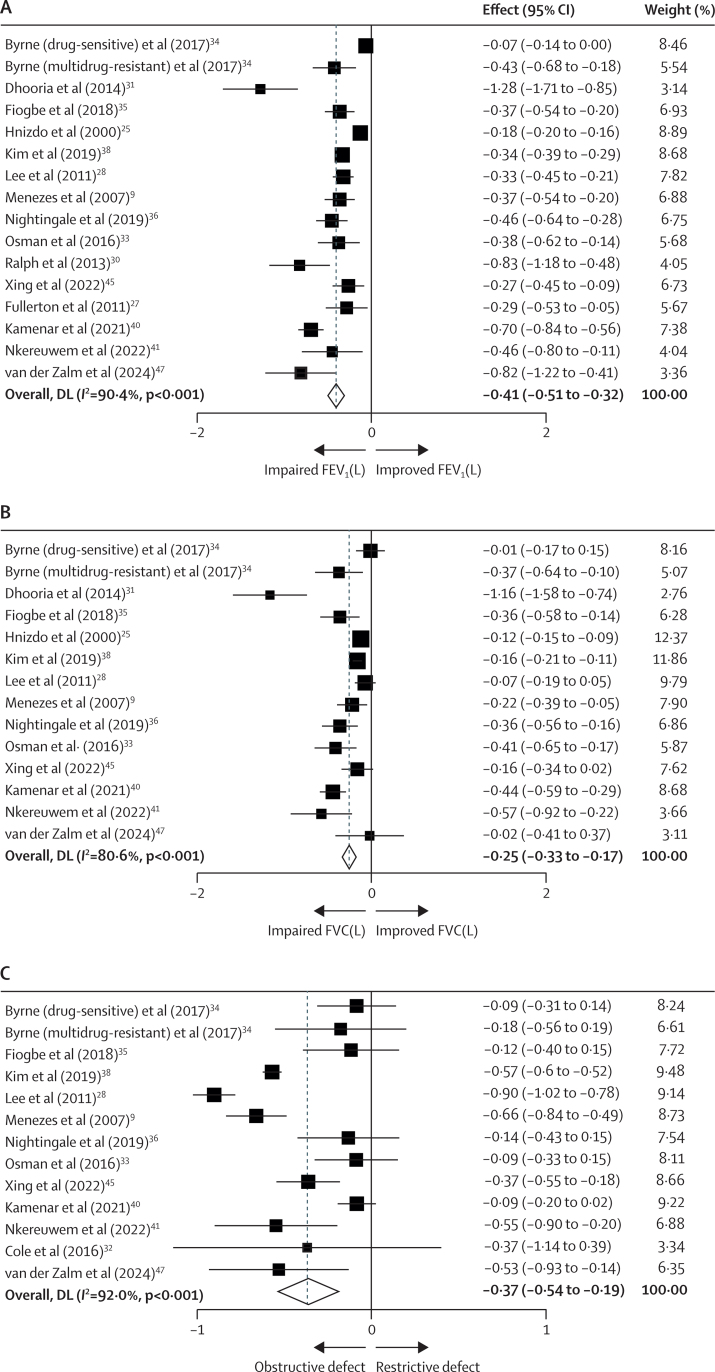
Effect of history of pulmonary tuberculosis on (A) FEV_1_, (B) FVC, and (B) FEV_1_/FVC ratio DL=DerSimonian and Laird method. FEV_1_=forced expiratory volume in 1 s. FVC=forced vital capacity. PTB=pulmonary tuberculosis. DS=drug sensitive. MDR=multidrug resistant. Weights are from random-effects model.

A total of 16 studies were included for FEV_1_ analysis and 14 for FVC, with varying levels of in-study adjustments or standardisation (table). Individuals with previous pulmonary tuberculosis had significantly lower pooled effect estimates for both FEV_1_ (–0·41 L, 95% CI –0·51 to –0·32, *I*^2^=90·4%) and FVC (–0·25 L, 95% CI –0·33 to –0·17, *I*^2^=80·6%) than controls (figure 2). Pooled SMD for FEV_1_/FVC ratio was –0·37 (95% CI –0·54 to –0·19, *I*^2^=92·0%; figure 2).

**Figure 3:**
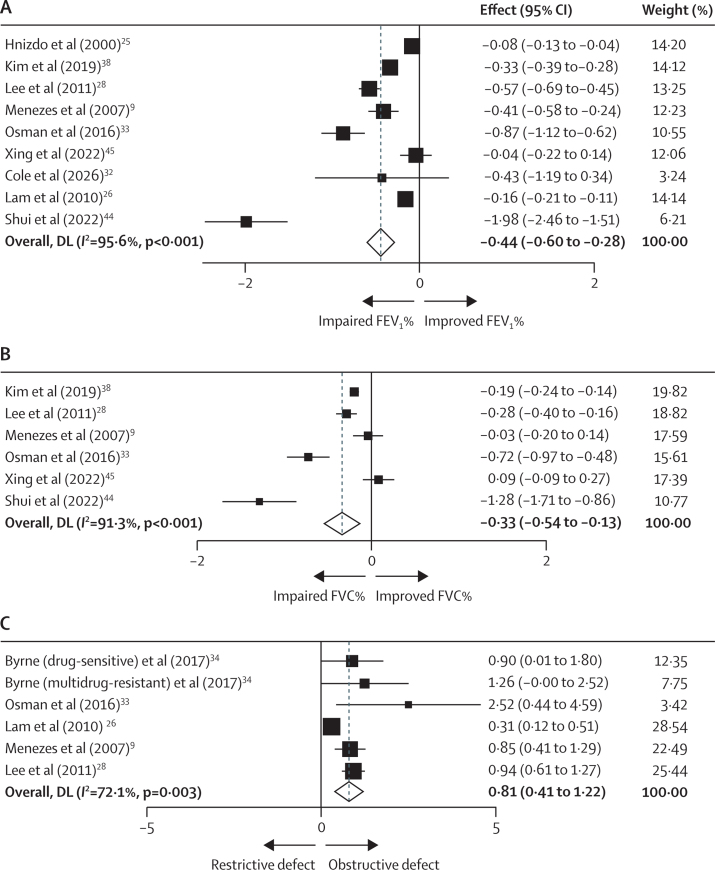
Effect of history of pulmonary tuberculosis on (A) FEV_1_%, (B) FVC%, and (C) log odds ratio of airflow obstruction DL=DerSimonian and Laird method. FEV_1_=forced expiratory volume in 1 s. FEV_1_%=FEV_1_ as a percentage of the predicted value. FVC=forced vital capacity. FVC%=FVC as a percentage of the predicted value. PTB=pulmonary tuberculosis. Airflow obstruction defined by an FEV_1_/FVC <0·7 or FEV_1_/FVC <lower limit of normal.

Nine studies included data on FEV_1_% and six on FVC% (table). Participants with previous pulmonary tuberculosis had significantly lower pooled estimates than controls: FEV_1_% was –0·44 (95% CI –0·60 to –0·28, *I*^2^=95·6%) and FVC% –0·33 (–0·54 to –0·13, *I*^2^=91·3%) (figure 3). Given that four studies reported adjusted odds ratios (OR) for airflow obstruction after pulmonary tuberculosis, post-hoc analysis was done to give a combined natural log OR of 0·81 (95% CI 0·41–1·22; figure 3). Back transformation gave a log OR of 2·25 (1·51–1·95). One study explicitly showed irreversible airflow obstruction.^26^ Regression analysis between each spirometric parameter and weighted mean age showed no significant correlation (appendix p 10).

Sensitivity analysis with the use of both fixed-effect and random-effect models (appendix pp 11–12) mostly showed higher pooled effect sizes with the latter, although both models reached statistical significance for all parameters. Leave-one-out analysis confirmed all studies remained within the 95% CIs, with no clear outliers (appendix p 13).

Heterogeneity was moderate to high across all analyses (*I*^2^ 72·1–95·6%; Figure 2, Figure 3). Subgroup analyses based on geographical region, adjustments, and study type conducted (appendix pp 14–15) did not significantly alter between-study heterogeneity. In instances when heterogeneity appeared to decrease, the number of included studies was substantially reduced, limiting the ability to draw definitive conclusions.

Funnel plot analysis and Egger's test for publication bias showed significant asymmetry with FEV_1_ (p=0·002) and FVC (p=0·01; appendix p 16). FEV_1_/FVC ratio did not show evidence of publication bias (p=0·2; appendix p 16). We did not have sufficient power to do statistical tests for bias for FEV_1_% and FVC% because a minimum of ten studies is necessary;^24^ however, visual inspection of FEV_1_% and FVC% funnel plots did not suggest small-study bias (appendix p 16).

Secondary outcomes collected showed that three studies found an association between chest x-ray lesions and lung function impairment.^25^, ^27^, ^34^ A west African study showed that previous tuberculosis was strongly associated with increased risk of chronic lung disease, outweighing typical risk factors in the region, such as smoking.^42^ One study showed no difference in inflammatory markers (ie, erythrocyte sedimentation rate, sputum and blood eosinophils, or C-reactive protein) between groups.^37^ However, in another study,^43^ elevated concentrations of matrix metalloproteinase-1 were observed in individuals with post-tuberculosis sequelae.

## Discussion

This systematic review and meta-analysis shows that a history of pulmonary tuberculosis significantly reduces objectively measured lung function compared with healthy controls. Overall, individuals with previous pulmonary tuberculosis had lower measured spirometric parameters than healthy controls—FEV_1_ (mean difference –0·41 L), FVC (mean difference –0·25 L), and FEV_1_/FVC ratio (SMD –0·37)—implicating a mainly obstructive ventilatory deficit in people who recovered. These findings are compelling when put into clinical context, as a decrease in FEV_1_ of 100 mL is considered clinically significant and a strong, independent predictor of cardiovascular and respiratory disease outcomes.^50^ These studies cross various WHO regions with varying tuberculosis incidence and income levels but consistently observe some form of pulmonary impairment following tuberculosis (appendix pp 4–7).

Our data are consistent with those of other population studies showing an increased risk of air flow obstruction in individuals who recovered from tuberculosis.^10^, ^51^ A meta-analysis from Byrne and colleagues^49^ found a pooled OR of 3·05 (95% CI 2·42–3·85) between past tuberculosis and COPD, with the strongest associations in countries with high tuberculosis incidence and in young people. Checkley's group further supports this finding in their study,^40^ which exclusively looked at low-income and middle-income countries (LMICs). The authors report an observed OR of 3·78 (2·87–4·98) between previous tuberculosis and airflow obstruction. Although we reported a modest adjusted pooled OR of 2·25, our primary meta-analysis dissects this relationship further with more detailed spirometric data.

We found that absolute FEV_1_ decreased more than did FVC, by 0·41 L and 0·26 L, respectively. Prospective studies, although scarce, suggest that the pattern of respiratory impairment varies through and beyond treatment completion.^35^, ^50^, ^51^ In pulmonary tuberculosis at diagnosis, Plit and colleagues^52^ found that FVC improved to a greater degree than FEV_1_ over the course of treatment. Nightingale and colleagues,^54^ having the longest follow-up period of 3 years, reported a third of individuals who recovered from tuberculosis experienced accelerated FEV_1_ decline. This observation implies that successful treatment might prevent restrictive sequelae to a greater degree than obstructive loss.

Although our data showed a lower FVC and FVC% decline, the effect size remains clinically and statistically significant (mean difference –0·25 L and SMD –0·33, respectively, p=0·0001 for both), suggesting that, although the net effect of pulmonary tuberculosis appears to be obstructive, a restrictive component is also present. Similarly, in a South African population study,^55^ a combined obstructive and restrictive defect was the most common functional outcome as a sequela of pulmonary tuberculosis in a South African population. Amaral and colleagues^51^ found that LMICs had the strongest association with both spirometric obstruction and restriction. It is apparent that current estimates of residual spirometric abnormalities after pulmonary tuberculosis vary widely according to the population.

The association between tuberculosis and lung function impairment is influenced by confounding risk factors such as biomass exposure, HIV, diabetes, and smoking.^56^ Smoking remains the highest risk factor for COPD globally, as well as a powerful risk factor for developing pulmonary tuberculosis.^56^ This bidirectional relationship might have influenced our findings; however, the data remained consistent even when adjusted for smoking (OR 2·25 for tuberculosis and airflow obstruction). Population studies in LMICs also indicate that pulmonary tuberculosis is a stronger risk factor for COPD than smoking.^8^, ^12^

These findings support the hypothesis that previous pulmonary tuberculosis is an independent risk factor for obstructive airways disease. In cumulative lung damage caused by smoking-related COPD, the extent of alveolar destruction and airway obstruction is slowly progressive, which makes this condition uncommon in younger people. On the other hand, pulmonary tuberculosis is primarily a disease of young adults, and the associated lung damage occurs during the acute disease process. This characteristic explains why pulmonary tuberculosis’ relative contribution to COPD is higher in the younger population, especially in tuberculosis endemic areas. This pattern has led to the term tuberculosis-associated obstructive lung disease.^11^, ^12^

The pathophysiology of the functional tuberculosis sequelae is speculative but is likely to be heterogeneous given the spectrum of clinical and radiological outcomes. Structural pulmonary changes resulting from aberrant tissue repair (eg, bronchovascular distortion, fibrosis, and pleural thickening) might explain airflow restriction.^3^ However, the mechanisms that drive airflow obstruction are poorly understood.^53^ This area of research is an important line of research as any pulmonary dysfunction can increase the risk of death from respiratory causes^3^ and contribute to the excess deaths observed in people who recover from tuberculosis.^5^, ^6^

The limitations of this study are in part related to the use of published data, with the validity of the results dependent on the conduct and reporting of the studies included. Second, a causal relationship between pulmonary tuberculosis and lung function impairment cannot be fully determined as the current evidence is primarily from cross-sectional and case–control studies. Pulmonary tuberculosis exposure was defined in some studies clinically, which could be another potential limitation if these individuals were not correctly diagnosed. However, all included studies were completed in regions of high tuberculosis incidence, so exposure risk is high (appendix pp 4–7). Furthermore, globally only 63% of pulmonary tuberculosis cases are diagnosed with microbiological confirmation,^1^ particularly in areas of high exposure where clinically diagnosed tuberculosis might be more common than in areas of low exposure.

Comparison of spirometric values across studies is challenging without adjusting for key variables. Although using percentage predicted values accounts for some variability, biases related to age, height, and ethnicity can remain, particularly at extremes. However, age-related bias might be mitigated by the predominantly younger study populations (<50 years; table). Although ethnic adjustments for lung function were not explicit, our findings align with those of other large single race reviews.^57^ Most studies also included some level of adjustment or standardisation for absolute spirometric values (table). Furthermore, OR analysis for airflow obstruction used the most adjusted values and still support our findings. Overall, despite these limitations, the observed effect size remains consistent and significant across all spirometric measures.

Although significant heterogeneity was observed with *I*^2^ (72·1–95·6%; Figure 2, Figure 3), this figure does not account for the other sources of statistical variability. Consequently, we compared *I*^2^ values with the use of both random effect and fixed-effect models (appendix pp 11–12). The latter method yielded lower effect estimates, suggesting true between-study variability.^24^ To address clinical and methodological heterogeneity, we conducted post-hoc subgroup analyses based on geographical region, adjustments, and study type (appendix pp 14–16). Although the direction of effect size remained consistent across subgroups, other reductions in heterogeneity were constrained by a substantial drop in study numbers (appendix pp 14–16). To further validate our findings, we performed leave-one-out analysis, confirming that all studies remained within the 95% CI, which indicates the robustness and reliability of our data, with no significant influence from a single study (appendix p 13). Residual heterogeneity might be partly attributed to study differences such as case selection, study quality, timing of lung function after treatment, and tuberculosis diagnostic criteria. However, tuberculosis itself is a highly complex disease with a spectrum of variable outcomes at both a population and individual level.^58^

A substantial research gap in this review is caused by the absence of data on plethysmography and gas transfer assays, which might be due to resource limitations. As lung pathologies often coexist (eg, emphysema and fibrosis) this can potentially lead to falsely low or preserved FVC readings.^59^, ^60^ In this instance, simple spirometry alone does not fully reveal residual respiratory impairments after pulmonary tuberculosis. Furthermore, potential effect modifiers, such as smoking history, HIV status, and other comorbidities, should also be consistently reviewed to understand the relationship between exposure and outcome.

The strengths of this review come with our adherence to PRISMA standards and the use of global multi-database searches, along with the input of expert groups, ensuring as much of the available literature as possible was captured. The decision to pool data was carefully considered based on comparable lung function outcomes (ie, FEV_1_, FVC) in comparison to healthy controls. Despite some variability across study populations, the core research question remained consistent between studies. This meta-analysis enhances understanding of respiratory deficits that are clinically and policy-relevant, providing granularity beyond broader spirometric definitions such as the Global Initiative for Obstructive Lung Disease criteria.^19^ The narrative synthesis further contextualizes these findings, offering insights into the factors driving the observed results.

In summary, these data present strong evidence that individuals affected by pulmonary tuberculosis have significant lung function loss with a mixed restrictive and obstructive picture with predominantly airflow obstruction compared with healthy populations. Previous population studies have observed a mainly obstructive pattern potentially due to their narrower focus on COPD. Our study has substantial implications for clinical practice and policy as currently post-tuberculosis lung disease remains an under-recognised global challenge, affecting tens of millions of people who have undergone pulmonary tuberculosis treatment worldwide. International guidelines need to include recommendations for medium and long-term follow-up of patients with pulmonary tuberculosis. This requirement will impose a large burden on both health and social care, which deserve greater integration into the WHO End Tuberculosis strategy. Finally, understanding the mechanisms that underpin this process might enable the development of host-directed therapies to limit or prevent the development of the chronic sequelae of pulmonary tuberculosis.

### Contributors

### Data sharing

Materials on characteristics of each study are available in the appendices. The extracted data used within our analysis are available on request to the authors.

**This online publication has been corrected. The corrected version first appeared at thelancet.com/lancetgh on July 22, 2025**

## Declaration of interests

We declare no competing interests.
