# Correction to *Lancet Glob Health* 2025; 13: e1020–29

**DOI:** 10.1016/S2214-109X(25)00224-4

**Published:** 2025-07-22

*Ratnakumar S, Hayward SE, Denneny EK, et al. Post-pulmonary tuberculosis lung function: a systematic review and meta-analysis.* Lancet Glob Health *2025; **13:** e1020–29*—In this Article, the spelling of author Sharenja Ratnakumar's name was incorrect. In the Summary, the funding line should have read “None”, and the Role of the funding source should have read “There was no funding source for this study.” The x-axis labels of figures 2 and 3 were incorrect. In paragraph 6 of the Results, the second and third sentences should have read, “Individuals with previous pulmonary tuberculosis had significantly lower pooled effect estimates for both FEV1 (–0·41 L, 95% CI –0·51 to –0·32, *I*^2^=90·4%) and FVC (–0·25 L, 95% CI –0·33 to –0·17, *I*^2^=80·6%) than controls (figure 2). Pooled SMD for FEV1/FVC ratio was –0·37 (95% CI –0·54 to –0·19, *I*^2^=92·0%; figure 2)”. In paragraph 7 of the Results, the third sentence should have read, “Given that four studies reported adjusted odds ratios (OR) for airflow obstruction after pulmonary tuberculosis, post-hoc analysis was done to give a combined natural log OR of 0·81 (95% CI 0·41–1·22; figure 3).” These corrections have been made as of July 22, 2025.

