# Supplementary material for: Post-pulmonary tuberculosis lung function: a systematic review and meta-analysis
Source: Lancet Glob Health. 2025 May 21;13(6):e1020–9. doi: 10.1016/S2214-109X(25)00105-6 (PMC12287636; doi:10.1016/S2214-109X(25)00105-6)

THE LANCET
Global Health

Supplementary appendix

This appendix formed part of the original submission and has been peer reviewed. We post it as supplied by the authors.

Supplement to: Ratnakumar S, Hayward SE, Denneny EK, et al. Post-pulmonary tuberculosis lung function: a systematic review and meta-analysis. *Lancet Glob Health* 2025; **13:** e1020–29.

Supplementary Material

S1: Search strategy

Search terms:

1. Tuberculosis
2. TB
3. MTB
4. 1 or 2 or 3
5. Lung adj4 test*
6. Pulmonary adj4 test*
7. Respiratory adj4 test*
8. PFT
9. Vital capacit*
10. FVC
11. Forced expiratory volume*
12. FEV1
13. Transfer factor*
14. Transfer coefficient*
15. Transfer capacit*
16. Diffusion factor*
17. Diffusion capacit*
18. TLCO
19. DLCO
20. KCO
21. Lung volume*
22. Flow volume loop*
23. Spiromet*
24. Peak expiratory flow*
25. Peak flow*
26. PEF
27. PEFR
28. Lung capacit*
29. Plethysmography
30. Residual capacit*
31. Residual volume*
32. 5 or 6 or 7 or 8 or 9 or 10 or 11 or 12 or 13 or 14 or 15 or 16 or 17 or 18 or 19 or 20 or 21 or 22 or 23 or 24 or 25 or 26 or 27 or 28 or 29 or 30 or 31
33. 4 and 32
34. limit 33 to yr="2000 -Current"

Note: 'n3' used in place of 'adj4' for CINAHL

Subject headings:

MEDLINE
1. exp Tuberculosis, Pulmonary
2. exp Mycobacterium tuberculosis
3. exp Respiratory Function Tests

EMBASE
1. exp Tuberculosis
2. exp Mycobacterium tuberculosis
3. exp Lung Function Test

CINAHL
1. Tuberculosis, Pulmonary
2. Mycobacterium tuberculosis
3. Respiratory Function Tests+

S2: Inclusion criteria, using PECOS framework

	**Inclusion criteria**	**Exclusion criteria**
**Population**	Any population in any geographic location	
**Exposure**	Cases are individuals with a prior diagnosis of active pulmonary TB disease (Eg. clinical, microbiological, self-reported or treatment history of PTB). Subjects will be included regardless of co-morbidities such as HIV.	
**Control**	Controls are healthy individuals without a prior diagnosis of active pulmonary TB disease.	The control group cannot have a selection bias towards a specific trait or comorbidity that could affect lung function performance (eg, significant respiratory disease, lower respiratory tract infection, hospitalisation, acute illness)

**Outcome**	Measurable effect on lung function, as identified through at least one lung function test<br><br>*E.g.* (but not limited to): Forced vital capacity (FVC), Forced expiratory volume (FEV1), Transfer/diffusion factor (TLCO or DLCO or KCO), Flow volume loops, Spirometry, Peak expiratory flow (PEF or PEFR), Residual volume (RV)	Included studies must report data that is disaggregated between TB cases and controls.<br><br>Research reporting on radiological or imaging evidence of fibrosis or markers of inflammation **only** (*i.e.* without a lung function test as well) will be excluded
**Study design**	Any study design other than prevalence studies or case reports without controls; pre-print papers or conference abstracts will be included	Non-original research where primary data are not reported *e.g.* commentaries, editorials, letters, reviews will be excluded
**Dates**	Papers published 1 Jan 2000- 13 Dec 2024	

	First Author (year)	WHO region	TB incidence	Income Group	Definition of PTB	Definition of controls	Ethnicity	Age PTB Cases	Age Control	Primary outcomes	Secondary outcomes
1	**Hnizdo (2000)[25]**	African	High	UMIC	Medical history (microbiological)	Miners who did not have past/active TB or pneumoconiosis	Black African	30-39	40-49	- Loss of lung function was highest within 6 months of diagnosis of TB and stabilised at 12 months.<br>- HIV status did not affect lung function loss<br>- AFO present in 18.4% with 1 episode of TB, 27.1% in 2 episodes, >3 episodes 35.2%	Average diagnosis of last episode of TB 4.6 years.
2	**Menezes (2007)[9]**	Americas	High and low	UMIC<br>HIC<br>UMIC<br>HIC<br>-	Medical history	>40years old. Analyses adjusted for sociodemographic variables, smoking, indoor and occupational exposure to pollution, and history of hospitalisation and comorbidities.	White, mixed, black, indigenous, Asian	56·6 ± 11·9	56·6 ± 11·9[§]	Prevalence of AFO[†] in past TB subjects was 30.7%.<br>Males had were more likely to have AFO[†] than females with past TB (OR 3.99 vs 1.21).	None recorded.
3	**Lam (2010)[26]**	Western Pacific	High	HIC	CXR scar	Included if they were capable of consenting, ambulatory, and not receiving treatment for life-threatening conditions.	Not described	61·9 ± 6·9	61·9 ± 6·9[§]	Prior TB associated with AFO[‡] (OR 1.37). No evidence of effect modification by smoking on the association between AFO and radiological evidence of prior TB.	Both radiological evidence and self-reported PTB were significantly associated with AFO (OR 1.43, 1.63 respectively)
4	**Fullerton (2011)**	African	High	LIC	Self-reported	Community members with no previous history of TB	Black African	39	39	Previous TB associated with reduced FEV1 (p=0.016)	Wood smoke and poverty contribute to reduced lung function and COPD is common in this population
5	**Lee (2011)[28]**	South-East Asian	High	HIC	CXR scar	Non-institutionalized Korean adults aged > 18 years.	Not described	53·3 ± 14	42·5 ± 14	Subjects with PTB CXR lesion had significantly lower FEV1, FEV1%, FVC%, FEV1/FVC than controls (all p <0001).	TB lesions on CXR were still associated with AFO[†] following adjustment for sex, age, and smoking history (OR 2.56).<br>Minimal TB lesions on CXR remained associated with AFO[†] (adjusted OR 3.13)
6	***Gomez Tejada (2012)[29]**	Americas	Low	UMIC	Medical history	Defined as 'normal'.	Not described	29 (21-29)	24 (21-33)	Past TB subjects had lower FEV1% compared to controls (p<0.05).<br>No significant bronchial responsivity between previous PTB and controls.	None recorded
7	**Ralph (2013)[30]**	South-East Asian	High	LMIC	Medical history (microbiological)	Local healthy controls were eligible if they were aged ≥18 years, gave written informed consent, and had no co-morbidities.	Papuan	28 (23-36·5)	26·5 (23·5-33)	FEV_1 lower in Past TB patients (p<0.0001) compared to controls.<br>27% of patients had severe lung function impairment	PTB subjects had lower 6MWT than controls (p=0.02).<br>57% of treated PTB subjects had respiratory symptoms.
8	**Dhooria (2014)[47]**	South-East Asian	High	LMIC	Medical history	Excluded active PTB, asthma, oral steroids, pregnancy, antihistamine use.	Not described	41·6 ± 14·0	40·0 ± 10·2	AFO was associated with Aspergillus sensitisation in subjects with PTB related fibrocavitary disease (OR 4.95)	Aspergillus sensitisation was found in 32% of PTB subjects with fibrocavitary disease. was associated with Aspergillus sensitisation on (OR 4.96)

9	**Cole (2016)**[31]	African	High	UMIC	Medical history	Healthy, self-reported HIV-negative adults with no history of PTB.	Black African	38 (21-65)	38 (21-65)[§]	FEV1 and FVC significantly lower in past TB participants.	N10one recorded.
10	**Osman (2016)**[32]	African	High	LIC	Medical history (microbiological)	Excluded acutely unwell, if had asthma or a recent pneumothorax.	Not described	44·0 ± 8·5	44·5 ± 8·6	Previous TB associated with chronic AFO[†] (12.4).	Chr11onic respiratory symptoms such as cough were strongly associated with a history of PTB after adjusting for potential confounders (OR 6.67).
11	**Byrne (2017)**[33]	Americas	High	UMIC	Medical history (microbiological)	Unexposed participants without TB were randomly selected from the same districts	Caucasian, African-american, Mestizo	30·0 (20·4-45·2)	37·6 (20·7 - 52·5)	Subjects with drug sensitive TB had higher FEV1 and FVC compared to controls (p non-significant). Risk of AFO[†] was higher in PTB group (OR 2.47, p=0.047)	No difference in respiratory symptoms (cough, wheeze, dyspnoea)), FeNO, or 6MWD between previous PTB and controls.
12	**Fiogbe (2018)**[34]	African	High	LMIC	Medical history (microbiological)	healthy volunteers (HIV–/TB–) in the general population	Black African	37 (30-47)	39 (33·5 - 52)	45% of previous PTB subjects had LFI (obstructive, restrictive, or mixed).	29.1% of cured PTB patients had an abnormal 6-MWT. Extent of initial radiological lesions, time between symptom onset and treatment, and female sex were independently associated with LFI in subjects with cured PTB.
13	**Nightingale (2018)**[35]	African	High	LIC	Self-reported	Excluded acutely unwell, non-permanent residents or pregnant.	Not described	43·8 ±17·8	43·8 ±17·8[§]	Previous TB was negatively associated with FEV1 (coefficient estimate -0.46) and FVC (-0.35).	Chronic respiratory symptoms were associated with prior PTB (OR 2.50).
14	***El Shoubargy (2019)**[36]	Eastern Mediterranean	Low	LMIC	Medical history	Excluded if diagnosis of COPD, asthma, overlap, rhinitis, heart failure, pregnancy, chronic debilitating disease.	Not described	39·35 ± 2·45	32·0 ± 2·62	FEV1 and FEV1% lower compared to controls (p =0.01, p=0.02) No correlation between time passed since PTB treatment completion and AHR.	More participants from PTB group had cough compared to controls. No difference in inflammatory markers (ESR, sputum eosinophilia, blood eosinophilia) between previous PTB subjects and controls.
15	**Kim (2019)**[37]	South-East Asian	High	HIC	Self-reported or CXR scar	Excluded active or previous PTB and if glomerular filtration rate <30 mL/min.	Not described	57·4 ± 0·5	48·7 ± 0·2	Respiratory dysfunction was more common in the past PTB group than in controls (restrictive pattern, 14.0% vs. 9.6%; obstructive pattern, 29.6% vs. 8.2%; both p<0.001).	Serum 25-hy-droxyvitamin D (25[OH]D) level was lower in past PTB participants than controls (p=0.013).
16	**Kamenar (2022)**[38]	Americas, South East Asia, African	High	LMIC	Self-reported	Excluded participants below age of 35 and above 95, active or previous history of TB, pregnant	Mixed across countries	54 (IQR 46.5-63.0)	54 (IQR 46.5-63.0)[§]	Previous tuberculosis disease was found to be an important risk factor for having COPD and worse lung function. There was evidence of effect modification by smoking status; smokers were less likely to have COPD in the setting of previous tuberculosis disease than non-smokers(p=0.02).	

17	**Fink (2022)**[40]	African	High	LMIC	Medical history	HIV negative used in analysis. Excluded following: pregnancy, eye, heart, chest, lung or abdominal surgery, myocardial infarction within past 3 months; 18heart rate or blood pressure, active TB.	Not specified, Nigerian community	41-50	31-40	History of TB was independently associated with increased risk of chronic lung disease	Suggest HIV and TB outweigh tobacco and domestic biomass fuel exposures as risk factors for chronic Lung disease in Nigeria
18	**Nkereuwem (2022)**[39]	African	High	LIC	Medical history (microbiological and clinical)	Age matched, no chronic lung disease	Not specified, Gambian community	8.9 (IQR 7.2–11.2	11.5 (8.0–13.7	Lung function impairment was present in 20/52 (38.5%) post--TB cases and 15/86 (17.4%) in the comparison group, p=0.009. Previous pTB and a history of chronic cough were significantly associated with the presence of lung function impairment (p=0.047 and 0.006 respectively). Forced expiratory volume in 1 s (FEV1), forced vital capacity (FVC) and FEV1/FVC z--scores were significantly lower in the post--TB cases compared with the comparison group (p= <0.001, 0.014 and <0.001, respectively)	The distribution of the self--reported physical health score, and parent--reported physical, emotional, psychological, social and total HRQoL scores were significantly lower in the post--TB cases compared with the comparison group.
19	**Shanmuga-sundaram (2022)**[41]	South-East Asia	High	LMIC	Medical history (microbiological)	Healthy controls – no difference in age, height or sex	Not specified, Indian outpatient clinic	46.20±10.05	37.50±9.95	Slow vital capacity (SVC), FVC, FEV_1, FEV_1/FVC, and peak expiratory flow were significantly lower in patients with post-TB sequelae (PTBS) compared to controls. SVC and FEV_1 were significantly less in PTBS as compared to post-TB patients without sequelae (PTBWS). Total airway impedance was significantly higher in PTBS as compared to PTBWS.	Serum MMP-1 level was significantly higher in PTBS as compared to other groups
20	**Shui (2022)**[42]	Western Pacific	High	HIC	Clinical diagnosis (bronchoscopy findings)	Age and BMI matched, non-smoking 'healthy' controls	Not specified, Chinese outpatient clinic	36.39±14.45	40±13.93	The maximal exercise capacity and PFT parameters of TBTB patients with central airway stenosis were impaired. Impaired exercise capacity correlated with the degree of central airway stenosis.	Lung volume ratio was a good predictor of exercise limitation in TBTB patients.
21	**Xing (2022)**[43]	Western Pacific	High	HIC	Medical history (self-reported) and	Exclusion criteria were active TB, myocardial infarction or	Not specified, residents	49.31±15.21	39.49±15.17	Post-TB is positively associated with pulmonary function impairment	Post TB associated with frequent respiratory symptoms.

					CXR scar	cerebrovascular accident during the previous 3 months; pregnancy; High heart rate or blood pressure, any condition that would impede the use of spirometry.	from Tibet and Xingjiang Uygur autonomous region			(airflow obstruction and small airways dysfunction).	
**22**	***Martinez (2023)**[44]	African	High	UMIC	Medical history (Microbiological and clinical)	No TB, 'remained healthy' during follow-up	Not specified, South African community	5	5[§]	Children with diagnoses of TB between 0 and 1 year of age had reduced time to peak tidal expiratory flow over total expiratory time (22.35% [95% CI, 24.86% to 20.17%]) and higher FeNO (2.88 ppb [95% CI, 0.57–5.19 ppb]) at 5 years. Children with diagnoses of TB between 1 and 4 years of age had impaired VT (29.32 ml [95% CI, 214.89 - 23.75 ml]) and time to peak tidal expiratory flow over total expiratory time (22.73% [95% CI, 25.45% to 20.01%]) at 5 years.	TB was associated with lower length-for-age (20.40 [95% CI, 20.68 to 20.11]), weight-for-age (20.30 [95% CI, 20.59 to 20.01]), and body mass index (20.54 [95% CI, 20.83 to 20.25]) z-scores at 5 years.
**23**	**Zalm (2024)**[45]	African	High	UMIC	Medical history (microbiological)	Healthy TB-exposed household contacts, with no prior PTB history.	Not specified, African community	16.4±2.0	13.4±2.5	Spirometry indices (FEV1, FVC, FEV1/FVC, TLC, DLCO) were lower in adolescents with tuberculosis compared to controls, after treatment completion.	Plethysmography in adolescents with tuberculosis showed that air-trapping was more common during treatment than in controls (12% vs 0%, respectively, p = 0.017); which improved following treatment completion. Adolescents with tuberculosis both during and after treatment completion walked a shorter distance than controls.

S3: Summary of all study characteristics

FEV_1=forced expiratory volume in 1 second; FVC=forced vital capacity; FEV_1% = FEV_1 as a percentage of predicted; FVC%= FVC as a percentage of predicted; VT = tidal volume; DLCO = diffusion capacity; PTB = pulmonary tuberculosis; UMIC=upper middle income country; LMIC= low middle income country; HIC=high income country; LIC=low income country; OR=odds ratio; AFO =airflow obstruction; COPD= chronic obstructive pulmonary disease; CXR = chest x-ray; FeNO= fraction of exhaled nitric oxide; 6MWD= 6-minute walk distance; 6MWT=6 minute walk time; ESR = erythrocyte sedimentation rate; AHR = airway hyperresponsiveness; LFI=lung function impairment; † AFO defined by FEV1/FVC<0.7; ‡ AFO defined by FEV1/FVC < lower limit of normal; [§]summarised data for whole population; *not included meta-analysis.

S4: Critical Appraisal (Joanna Briggs Institute Critical Appraisal Tool)

a) Checklist questions

	Cohort studies	Case-control studies	Cross-sectional studies
1	Were the two groups similar and recruited from the same population?	Were the groups comparable other than the presence of disease in cases or the absence of disease in controls?	Were the criteria for inclusion in the sample clearly defined?
2	Were the exposures measured similarly to assign people to both exposed and unexposed groups?	Were cases and controls matched appropriately?	Were the study subjects and the setting described in detail?
3	Was the exposure measured in a valid and reliable way?	Were the same criteria used for identification of cases and controls?	Was the exposure measured in a valid and reliable way?
4	Were confounding factors identified?	Was exposure measured in a standard, valid and reliable way?	Were objective, standard criteria used for measurement of the condition?
5	Were strategies to deal with confounding factors stated?	Was exposure measured in the same way for cases and controls?	Were confounding factors identified?
6	Were the groups/participants free of the outcome at the start of the study (or at the moment of exposure)?	Were confounding factors identified?	Were strategies to deal with confounding factors stated?
7	Were the outcomes measured in a valid and reliable way?	Were strategies to deal with confounding factors stated?	Were the outcomes measured in a valid and reliable way?
8	Was the follow up time reported and sufficient to be long enough for outcomes to occur?	Were outcomes assessed in a standard, valid and reliable way for cases and controls?	Was appropriate statistical analysis used?
9	Was follow up complete, and if not, were the reasons to loss to follow up described and explored?	Was the exposure period of interest long enough to be meaningful?	
10	Were strategies to address incomplete follow up utilized?	Was appropriate statistical analysis used?	

11	Was appropriate statistical analysis used?		

b) Critical appraisal results

First author	Checklist used (cohort, case control, etc)	1	2	3	4	5	6	7	8	9	10	11	Total (sum of Y)
Byrne	Cohort	Y	Y	Y	Y	Y	N	Y	NA	NA	NA	Y	7/11
Cole	Cross-sectional	Y	N	N	Y	Y	N	Y	Y				5/8
Dhooria	Case-control	Y	Y	N	Y	Y	Y	Y	Y	Y	Y		9/10
El Shourbagy	Case-control	Y	Y	Y	Y	Y	N	N	Y	U	Y		7/10
Fiogbe	Cross-sectional	Y	Y	Y	Y	Y	Y	Y	Y				8/8
Fink	Cross-sectional	Y	Y	N	N	Y	Y	Y	Y				6/8
Fullerton	Cross-sectional	N	N	Y	N	Y	N	Y	Y				4/8
Gomez Tejada	Case-control	N	N	Y	Y	N	N	N	Y	U	Y		4/10
Hnizdo	Cohort	Y	Y	Y	Y	Y	N	Y	Y	NA	NA	Y	8/11
Kamenar	Cross-sectional	Y	Y	Y	Y	Y	Y	Y	Y				8/8
Kim	Cross-sectional	Y	Y	Y	Y	Y	Y	Y	Y				8/8
Lam	Cross-sectional	Y	Y	Y	Y	Y	Y	Y	Y				8/8
Lee	Cross sectional	N	N	Y	Y	Y	Y	Y	Y				6/8
Martinez	Cohort	Y	Y	Y	Y	Y	Y	Y	U	Y	Y	Y	10/11
Menezes	Cross-sectional	N	Y	Y	Y	Y	Y	Y	Y				7/8
Nightingale	Cross-sectional	U	Y	Y	Y	Y	Y	Y	Y				7/8
Nkereuwem	Cross-sectional	Y	Y	Y	Y	Y	N	Y	Y				7/8
Osman	Case-control	Y	Y	U	Y	N	Y	Y	Y	Y	Y		8/10
Ralph	Case control	N	Y	N	Y	U	Y	Y	Y	U	Y		6/10
Shanmugnathan	Case-control	Y	Y	U	Y	U	Y	Y	Y	NA	Y		7/10
Shui	Case-control	N	Y	Y	U	U	Y	N	Y	NA	Y		5/10
Xing	Cross-sectional	N	Y	Y	Y	Y	N	Y	Y				6/8
Zalm	Cohort	Y	Y	Y	Y	Y	NA	Y	Y	Y	U	Y	9/10

S5: Regression analysis: weighted mean age versus lung function

Parameter	Coefficient	Standard error	P value
FEV_1	0.005	0.006	0.4
FVC	0.0004	0.007	0.9
FEV_1/FVC	-0.002	0.006	0.8
FEV1%	0.031	0.249	0.252
FVC%	0.040	0.033	0.285

S6: Sensitivity analysis a) random- and fixed-effects models

Parameter	Studies included	Fixed or random effects	Pooled effect size	Heterogeneity (I^2)	P value
FEV_1	16	random	-0.41	90.4%	<0.0001
FEV_1	16	fixed	-0.22	90.4%	<0.0001

Parameter	Studies included	Fixed or random effects	Pooled effect size	Heterogeneity (I^2)	P value
FVC	14	random	-0.25	80.6%	<0.0001

Parameter	Studies included	Fixed or random effects	Pooled effect size	Heterogeneity (I^2)	P value
FVC	14	fixed	-0.14	80.6%	<0.0001
FVC_1/FVC	13	random	-0.37	92.0%	<0.0001
FEV_1/FVC	13	fixed	-0.49	92.0%	<0.0001

Parameter	Studies included	Fixed or random effects	Pooled effect size	Heterogeneity (I^2)	P value
FEV_1%	9	random	-0.44	95.6%	<0.0001
FEV_1%	9	fixed	-0.21	95.6%	<0.0001

Parameter	Studies included	Fixed or random effects	Pooled effect size	Heterogeneity (I^2)	P value
FVC%	6	random	-0.33	91.3%	0.002

FVC%	6	fixed	-0.21	91.3%	<0.0001

S6: Sensitivity analysis b) Leave one out analysis i) FEV_1, ii) FVC, iii) FEV_1/FVC, iv) FEV_1%, v) FVC%

i)

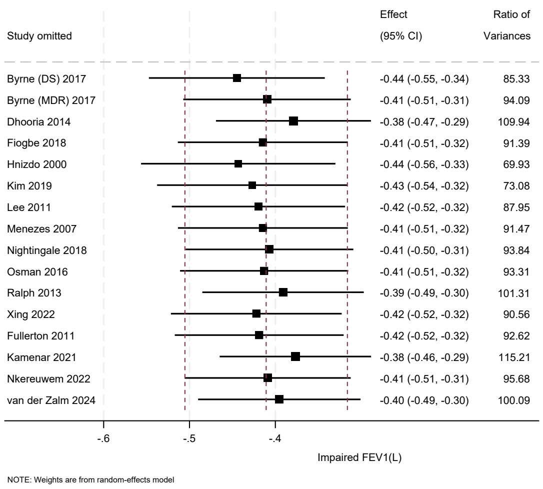

ii)

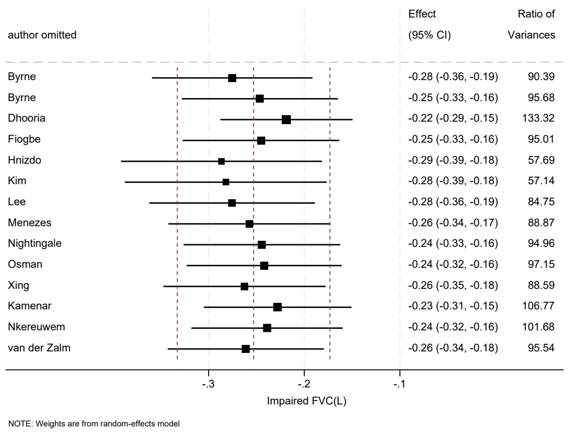

iii)

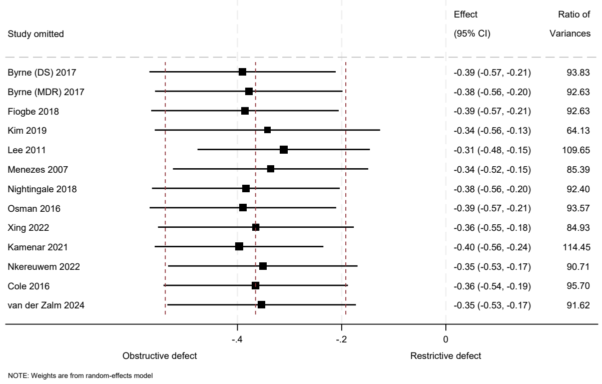

iv)

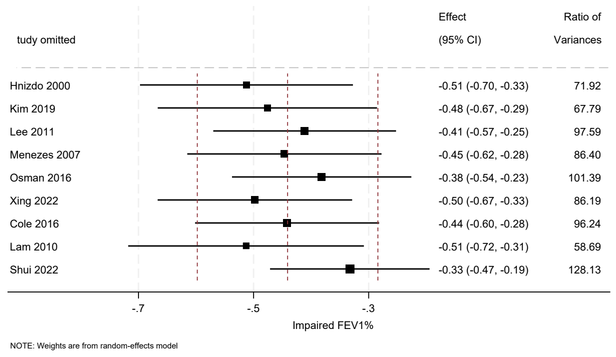

v)

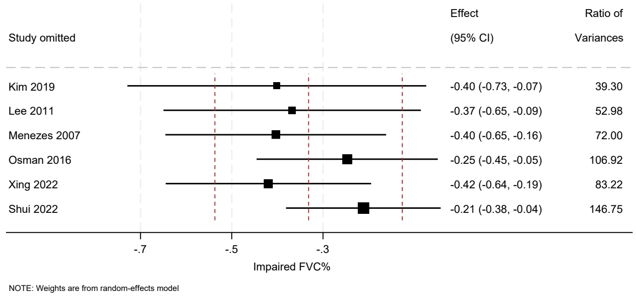

S7: Subgroup analysis by geographical region, adjustment and study type

**FEV_1 Subgroup**<br>**(n=15)**	Studies<br>included	Pooled effect<br>size (MD)	Heterogeneity<br>(I^2)	P value
African	8	-0.44	90.6%	<0.0001
Non-African	8	-0.41	89.8%	<0.0001
**FVC Subgroup**	Studies	Pooled effect	Heterogeneity	P value
Adjusted<br>**(n=13)**	12<br>included	-0.47<br>size (MD)	91.3%<br>(I^2)	<0.0001
Non-adjusted	4	-0.34	0%	<0.0001
African	7	-0.32	83.7%	<0.0001
Cross-sectional	9	-0.40	67.1%	<0.0001
Non-African	7	-0.22	79.6%	0.001
**FEV_1/FVC**	Studies	Pooled effect	Heterogeneity	P value
Adjusted<br>**Subgroup (n=12)**	10<br>included	-0.35<br>size (SMD)	86.0%<br>(I^2)	<0.0001
Non-adjusted	4	-0.15	0%	<0.0001
African	7	-0.20	42.2%	0.004
Cross-sectional	8	-0.26	72.4%	<0.0001
Non-African	6	-0.49	91.1%	<0.0001
**FEV_1% Subgroup**	Studies	Pooled effect	Heterogeneity	P value
Adjusted<br>**(n=9)**	9<br>included	-0.17<br>size (SMD)	24%<br>(I^2)	0.001
Non-adjusted	4	-0.63	90.6%	<0.0001
African	3	-0.46	94.8%	0.161
Cross-sectional	9	-0.43	93.5%	<0.0001
Non-African	6	-0.45	95.3%	<0.0001
Chinese/Korean	5	-0.47	96.2%	<0.0001
Non-Chinese/Korean	4	-0.44	93.9%	0.026
Adjusted	4	-0.84	96.9%	0.034
Non-adjusted	5	-0.30	93.0%	<0.0001

Cross-sectional	6	-0.31	91.3%	<0.0001

**FVC% Subgroup (n=6)**	Studies included	Pooled effect size (SMD)	Heterogeneity (I^2)	P value
Chinese/Korean	4	-0.31	91.8%	0.011
Non-Chinese/Korean	2	-0.37	95.1%	0.282
Adjusted	2	-0.97	80.1%	0.001

Non-adjusted	4	-0.12	78.8%	0.067
Cross- sectional	4	-0.12	78.8%	0.067

S8: Funnel plot and Eggers test for a) FEV_1, b)FVC, c)FEV_1/FVC, d)FEV_1%, e)FVC%

a) FEV_1

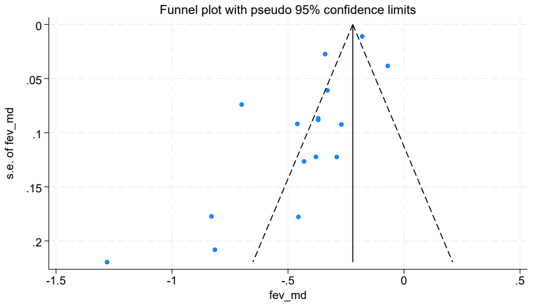


```
Number of studies =  16                              Root MSE      =   2.343

    Std_Eff   Coefficient  Std. err.       t    P>|t|     [95% conf. interval]

      slope    -.1525971   .0282506     -5.40   0.000    -.2131887   -.0920055
       bias    -2.881378   .7585047     -3.80   0.002    -4.508209   -1.254548

Test of H0: no small-study effects          P = 0.002
```

d) FEV_1%

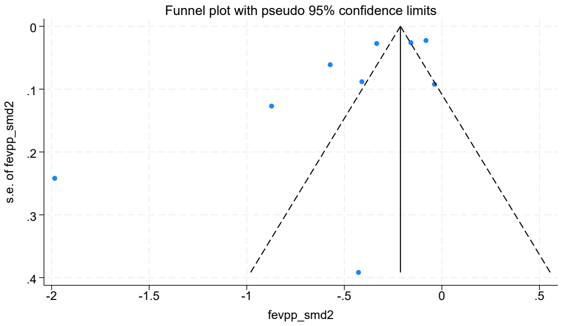

b) FVC

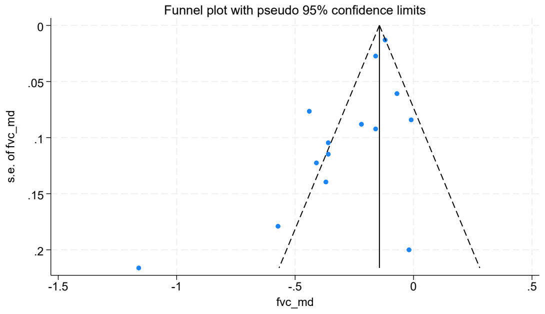


```
Number of studies =  14                              Root MSE      =    1.79

    Std_Eff   Coefficient  Std. err.       t    P>|t|     [95% conf. interval]

      slope    -.0934979   .0258265     -3.62   0.004     -.149769   -.0372267
       bias    -1.889071   .6344066     -2.98   0.012    -3.271324   -.5068179

Test of H0: no small-study effects          P = 0.012
```

e) FVC%

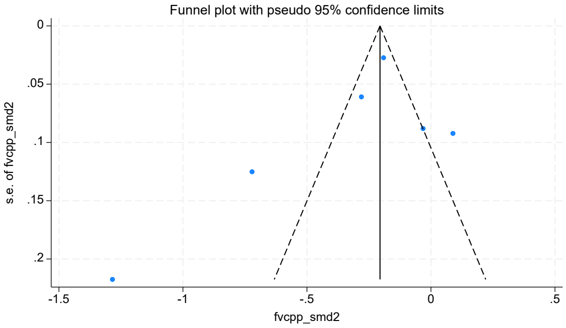

c) FEV_1/FVC

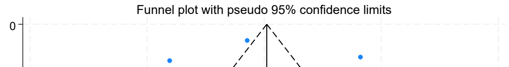

Supplement: Supplementary appendix [file mmc1.pdf]
